# Zika Virus in West Africa: A Seroepidemiological Study between 2007 and 2012

**DOI:** 10.3390/v12060641

**Published:** 2020-06-13

**Authors:** Serena Marchi, Simonetta Viviani, Emanuele Montomoli, Yuxiao Tang, Adele Boccuto, Ilaria Vicenti, Maurizio Zazzi, Samba Sow, Aldiouma Diallo, Olubukola T. Idoko, Niranjan Bhat, Claudia Maria Trombetta

**Affiliations:** 1 Department of Molecular and Developmental Medicine, University of Siena, 53100 Siena, Italy; serena.marchi2@unisi.it (S.M.); simoviviani56@gmail.com (S.V.); emanuele.montomoli@unisi.it (E.M.); 2 VisMederi srl, 53100 Siena, Italy; 3 PATH, Seattle, WA 98121, USA; yuxiaotang@path.org (Y.T.); nbhat@path.org (N.B.); 4 Department of Medical Biotechnologies, University of Siena, 53100 Siena, Italy; adele.boccuto@gmail.com (A.B.); ilariavicenti@gmail.com (I.V.); maurizio.zazzi@gmail.com (M.Z.); 5 Centre for Vaccine Development (CVD), Bamako BP 251, Mali; ssow@som.umaryland.edu; 6 IRD, VITROME, Dakar BP 1386 CP 18524, Senegal; aldiouma.diallo@ird.fr; 7 Medical Research Council Unit, The Gambia at London School of Hygiene and Tropical Medicine, Banjul P.O. Box 273, The Gambia; bukkyidoko@gmail.com

**Keywords:** Zika virus, immunity, seroprevalence, epidemiology, West Africa

## Abstract

According to the World Health Organization, the entire African continent is at risk of a Zika outbreak. To increase data availability on the epidemiology of Zika virus circulation in Africa, we evaluated the immunity to Zika virus in a selected cohort of subjects from West Africa between 2007 and 2012. Human serum samples were collected in 2007 and in 2011/2012 from a cohort of 2–29-year-old subjects from Mali, Senegal, and The Gambia. A sample that tested positive by Zika virus IgG ELISA and by Zika virus microneutralization test was defined as positive. In 2007, the highest prevalence was 21.9%, found in Senegal among 18–29-year-old subjects. In 2011/2012, the highest prevalence, 22.7%, was found still in Senegal, but in 11–17-year-old subjects. During both study periods, the lowest prevalence was found in Mali, where few positive cases were found only in 18–29-year-old subjects. The Gambia showed an intermediate prevalence. In the three countries, prevalence was strongly associated with increasing age. This study contributes to understanding Zika virus circulation within three different ecological and demographic contexts with scarce or no data currently available. Results showed that Zika virus circulated actively in West Africa between the period 2007 and 2011/2012, but with some geographic specificity.

## 1. Introduction

Zika virus (ZIKV) is a *Flavivirus* transmitted to humans mainly by *Aedes* mosquitoes that was first isolated in Uganda in 1947 [1,2]. Serological data suggest that ZIKV transmission has occurred among humans, animals and mosquitoes throughout tropical Africa for more than 70 years; however, ZIKV epidemics were never reported, and fewer than 20 human infections were recorded [3] between its isolation and the first large epidemic, occurring in Micronesia in 2007 [4]. ZIKV gained new attention after its spread in the Pacific and then to the Americas [5], and in recent years serological studies were conducted in Africa, documenting the presence of ZIKV antibodies in humans [6,7,8,9]. Moreover, a ZIKV outbreak that occurred in Gabon in 2007 was retrospectively identified [10]. Further, in October 2015, an outbreak of more than 7000 suspected cases was reported in Cape Verde with the identification of the Asian lineage of ZIKV [11,12]. More recently, transmission of ZIKV has been reported in Guinea-Bissau (African lineage) and Angola (Asian lineage) [13,14].

ZIKV infection in humans is asymptomatic in about 80% of cases [4]. When symptoms occur, they are typically mild, self-limiting and similar to other arboviral infections. Commonly reported symptoms include macupapular rash, low-grade fever, arthralgia, myalgia, headache, and conjunctivitis [1]. During the recent pandemic, ZIKV infections were associated with neurological complications in adults (myelitis, encephalitis, Guillain-Barré syndrome) and auditory, ocular, and neurological malformations (including microcephaly) in newborns, whose mothers contracted the virus during pregnancy [2]. Moreover, sexual transmission of ZIKV has been described, with ZIKV RNA detection in semen up to 6 months after symptoms onset [15]. The overlap of clinical manifestations of ZIKV in adults with other arboviruses and the high levels of flaviviral cross-reactivity can potentially lead to misdiagnosis and underestimation of circulation in these areas where such viruses co-circulate [2]. In fact, historical evidence of ZIKV infection depend on the serological method used, that may be affected by cross-reactions with other flaviviruses, especially in these high-risk areas. It is therefore likely that sporadic human cases of ZIKV infection have occurred for decades but were unrecognized.

The population living in endemic areas and repeatedly exposed to ZIKV may acquire immunity against the virus, thus leading to a reduced risk of developing an outbreak. According to serological studies, the distribution of exposure to ZIKV infections suggests the acquisition of immunity related to increasing age [16,17]. It is therefore possible that countries regarded as high-risk due to past reports of cases and serological evidence, may also be those whose populations have immunity, and therefore reduced risk. On the other hand, younger previously unexposed populations may remain vulnerable and constitute an immunity gap for when circulation reoccurs; thus, acquired immunity may have limited impact in averting the consequences of ZIKV infection on pregnant women and foetal health [16]. According to the World Health Organization (WHO), all the countries in the African region are at risk of developing a ZIKV outbreak [16]. The potential for ZIKV transmission for each country was assessed by WHO using information on current and past reports of ZIKV infections in humans, the presence of *Aedes aegypti* as a competent vector and the confirmed transmission of other *Aedes*-borne viruses, such as dengue (DENV) and chikungunya (CHIKV) viruses, as evidence of sufficient vector density and efficiency. In West Africa, three countries with different ZIKV transmission potential were identified: Senegal, with present local cases; Mali, with only serological evidence; The Gambia, with only local cases of DENV and CHIKV infections [16].

A ZIKV phylogenetic study indicated the central role played by Senegal in the evolution and genotypic divergence of ZIKV, identifying two different African lineages (African lineage 1 and African lineage 2) and suggesting West Africa as the geographic origin of ZIKV with spread to Asian countries and Pacific islands [18]. To date, limited data are available on the prevalence of ZIKV in West African populations. The aim of this study was to evaluate the prevalence of ZIKV antibodies in a selected sample of West African populations aged 2–29 years from Senegal, The Gambia and Mali in 2007, and again in the same subjects between 2011 and 2012.

## 2. Materials and Methods

Human serum samples were collected during one of the clinical trials performed within the framework of the Meningitis Vaccine Project (MVP) [19]. The clinical trial was performed in 2007 in Mali, The Gambia, and Senegal to evaluate the safety and immunogenicity of MenAfriVac^®^ vaccine (Serum Institute of India, Pune, India) in comparison to a licensed comparator, Meningococcal Vaccine, in 2–29-year-old participants [20]. Acute disease (with or without fever) at the time of enrollment, administration of immunoglobulins and/or any blood products in the previous 30 days, administration of immunosuppressants or other immune-modifying agents in the previous 90 days, immunodeficiency or serious chronic illness, pregnancy or lactation were among the exclusion criteria. For each blood sample collected, a thick smear was examined for malaria parasitemia before vaccination. To evaluate antibody persistence to MenAfriVac^®^ vaccine (Serum Institute of India, Pune, India), blood samples from the same study participants were collected between 2011 and 2012 [21]. The study was conducted under the International Council for Harmonization of Technical Requirements for Pharmaceuticals for Human Use Good Clinical Practice (ICH-GCP) with identifier ISRCTN87739946.

**Figure 1:**
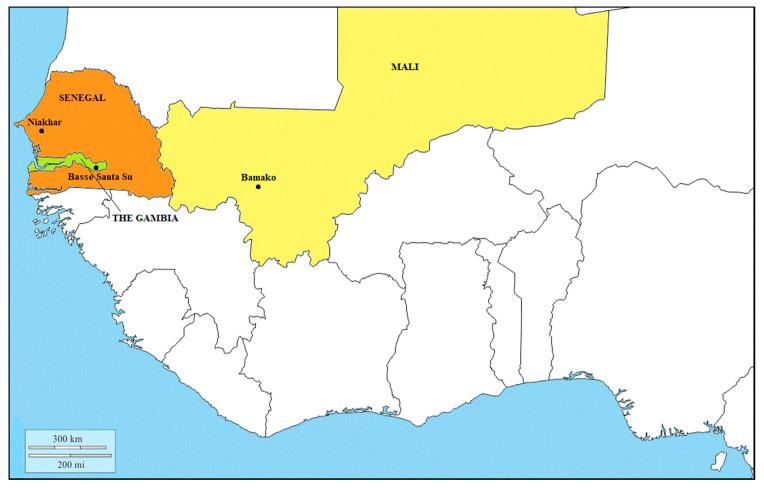
West African region, study sites in Mali (yellow), Senegal (orange) and The Gambia (green).

A total of 871 available samples were collected before vaccination between August and October 2007 from subjects of both sexes and aged 2–29 years in three different study sites: Bamako, Mali; Niakhar, Senegal; Basse Santa Su, The Gambia (Figure 1).

Out of the 650 samples collected between November 2011 and April 2012 at the same study sites and part of this study, 627 belonged to the same subjects of the 2007 cohort. After the study’s completion, samples and corresponding data were banked and anonymized. The present study was approved by Ethic Committees in Mali, Senegal and The Gambia (Senegal: 0-000124, released 14 September 2017; Mali: 240/CVD-Mali/CNAM, released 12 September 2017; Gambia: SCC1574v1.1, released 16 October 2017).

For each study site, samples were stratified by age group (2–10, children; 11–17, adolescents; 18–29 year-old, young adults) and sex, as shown in Table 1.

All sera were tested for presence of ZIKV IgG antibodies by commercial ELISA (Euroimmun^TM^, Lübeck, Germany), following the manufacturers’ instructions. Samples were considered positive when the ratio between the optical density (OD) of the sample and that of the calibrator was >1.1, negative when the ratio was <0.8.

All sera with borderline or detectable ZIKV IgG ELISA antibodies were tested by an in-house ZIKV microneutralization test (MNT) [22]. MNT was performed in a 96-well format based on cytopathic effect (CPE), using the ZIKV strain UVE/ZIKV/1947/UG/MR766 obtained from the European Virus Archive (EVAg, Genbank reference DQ859059), Vero E6 cells (ATCC^®^ CRL-1586™) and serum dilutions from 1:10 to 1:640. Samples with titer ≥1:40 were considered positive [23] and surrogate threshold of immunity.

For the purpose of this study, we defined as positive to ZIKV a subject whose sample tested positive to ZIKV IgG ELISA and to ZIKV MNT. Statistical analyses were performed using SAS^®^ software version 9.4 (SAS Institute Inc., Cary, NC, USA). Sex- and age-specific prevalence rates and seroconversion rates of samples collected in 2007 and 2011/2012 from the same subjects were calculated for each study site along with the corresponding exact two-sided 95% confidence interval (CI) obtained using the Clopper–Pearson method. The association of prevalence rate with age groups and study sites during each study period was examined by a logistic regression model, adjusting for sex and malaria parasitaemia test result. The model for the later study period was additionally adjusted for the meningococcal group A (MenA) specific antibody titers measured by serum bactericidal antibody assay with rabbit complement (rSBA), 28 days following MenA vaccination, to explore any interaction between the immune response to MenAfriVac^®^ (Serum Institute of India, Pune, India) vaccination and immunity against ZIKV. Logistic analysis was also used to investigate the association of the seroconversion rate with age groups and study sites adjusting for sex. A significance level of 0.05 was used for the two-sided statistical tests.

## 3. Results

Out of 1521 samples, 142 showed borderline or detectable ZIKV IgG ELISA. A total of 95 samples out of 142 also tested positive to ZIKV-MNT and were considered positive for the purpose of this study.

**Figure 2:**
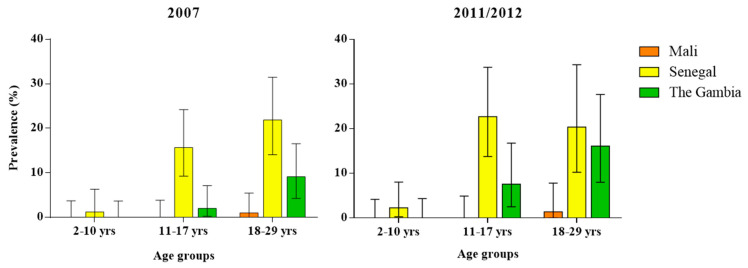
Anti-ZIKV MNT antibody prevalence in Mali, Senegal and The Gambia, by age group: (**left**) in 2007 and (**right**) in 2011/2012.

In 2007, 13.4% (95%CI 9.65–17.90) of all samples in Senegal were positive to ZIKV, followed by The Gambia (3.7%, 95%CI 1.87–6.55) and Mali (0.3%, 95%CI 0.01–1.90). The highest prevalence was found in Senegal in 18–29 and in 11–17-year-old subjects, with rates of 21.9% (95%CI 14.08–31.47) and 15.7% (95%CI 9.24–24.22), respectively (Table 2). In 2011/2012, the highest prevalence of all ZIKV positive samples continued to be in Senegal (13.7%, 95%CI 9.4–19.14), followed by The Gambia (7.1%, 95%CI 4.03–11.45) and Mali (0.4%, 95%CI 0.01–2.42). The 11–17-year-old subjects in Senegal had the highest seroprevalence to ZIKV, with a rate of 22.7% (95%CI 13.79–33.79), followed by the 18–29-year-olds, with 20.4% (95%CI 10.24–30.34) and with 16.1% (95%CI 8.02–27.67) in the same age group in The Gambia. In both study periods, no ZIKV positive subjects were found among the 2–10- and 11–17-years-old age groups in Mali, and the 2–10-years-old age group in The Gambia (Table 2, Figure 2).

In both study periods, differences in ZIKV prevalence rates between study sites were statistically significant (*p* < 0.0001 for 2007 and *p* = 0.001 for 2011/2012). No significant differences were found between males and females, while a clear trend of increasing ZIKV positivity was found with increasing age (*p* = 0.0004) (Table 2, Figure 2). No statistically significant association was found between ZIKV prevalence rate and malaria parasitaemia test result in either study period. The MenA rSBA titer measured at 28 days post-vaccination was not shown to be significantly associated with ZIKV prevalence rate in the 2011/2012 study period.

When comparing serologic results between 2007 and 2011/2012 within the same subject, the highest seroconversion rate to ZIKV was observed in Senegal (4%, 95%CI 1.76–7.81) with seroconversion in six subjects aged between 11–17-years-old and one subject in each of the 2–10 and 18–29-year-old age groups, followed by The Gambia with seroconversion in six (2.9%, 95%CI 1.07–6.17) subjects, three in each 11–17- and 18–29-year-old age groups, and by Mali with only one subject (0.5%, 95%CI 0.01–2.5) seroconverted in the 18–29-year-old age group (Table 3).

The differences in seroconversion rates among sites and among age groups were not statistically significant. No statistically significant differences were found between males and females either.

Persistence of antibodies between 2007 and 2011/2012 showed that 19 subjects (52.8%, 95%CI 37.0–68.02) over a total of 36 found positive in 2007 maintained the same MNT titer in 2011/2012. For seven subjects (19.4%, 95%CI 9.45–35.33) an increase in the MNT antibody titer was observed, while for 10 subjects (27.8%, 95%CI 15.7–44.14) a decrease in the MNT titer occurred. Among these 10 subjects, six (16.7%, 95%CI 7.49–32.27) were found to be negative in the 2011/2012 sample (Table 4) or with a ZIKV MNT titer < 1:40.

Differences or trends in increasing or decreasing MNT titer by age groups or sex were not statistically significant.

## 4. Discussion

The present study provides data on the circulation of ZIKV in West Africa during the years of its emergence in the Pacific region [4]. Results of this study indicate that in 2007 and 2011/2012, ZIKV was present and actively circulating in Senegal and The Gambia and yet virtually absent in Mali.

In Senegal, the overall prevalence of seropositivity to ZIKV was 13%, and was higher in subjects older than 11 years during both study periods. These findings confirm previous WHO reports, noting the presence of local cases of ZIKV infection in the region [16]. Samples were collected in Niakhar, a rural area that most likely allows exposure to both urban and sylvan mosquitoes. A study conducted in Senegal on samples collected from 1992 to 2016 [8] showed that ZIKV IgM prevalence among febrile patients was between 5% and 7.5%, demonstrating continuous transmission of ZIKV in Senegal over two decades. In our study, 4% of subjects from Senegal seroconverted for ZIKV between 2007 and 2011/2012, confirming the findings of Herrera et al. [8].

In our study, ZIKV prevalence in The Gambia was somewhat lower than in Senegal with no positive samples in children in both study periods. As for Senegal, samples from The Gambia were collected in a rural village with similar climatic and ecological conditions, including the presence of competent vectors, as in Niakhar. It is therefore difficult to explain the differences in ZIKV prevalence between the two areas. We could hypothesize that probably there is a difference in the *Aedes* population in The Gambia for local ecological reasons, or a heterogeneity due to the time and place of sampling. To our knowledge, this is the first report on the presence of ZIKV in humans from The Gambia, where the WHO report indicates only the presence of DENV and CHIKV as local cases [16].

Our findings show that ZIKV is virtually absent in Mali, with only a few positive subjects in the 18–29-year-old age group. One possible explanation is that, despite the urban environment being extremely favorable to the survival of *Aedes aegypti* in Bamako, transmission of ZIKV is reduced and almost absent compared to the rural study locations.

One interesting observation is that all the three countries included in this study implemented a similar malaria control program during the study period, leading to a steady decline in malaria cases up to a pre-elimination stage [24,25]. Many strategies erected against malaria and its vector, e.g., long-lasting insecticide-treated nets, are unlikely to significantly impact *Aedes* populations, because of biological and behavioral differences [26]. In addition, in our study no interaction between the presence of malaria parasitemia and ZIKV antibody presence was observed, suggesting that co-infections are common in sites where exposure to both *Anopheles* and *Aedes* vectors co-exist.

Our study confirms that ZIKV antibody acquisition is strongly related to increasing age. The increasing prevalence concurrent with age suggests that the population is consistently exposed to ZIKV throughout life. Furthermore, higher rates observed in the elderly may be the result of an earlier discrete exposure, followed by a decrease in ZIKV circulation that left younger cohorts unexposed. According to the number of seroconverted subjects between 2007 and 2011/2012, it is unlikely that a ZIKV epidemic had occurred in any of the three study locations, although some underestimation of new cases in 2011/2012 may have occurred, given the number of subjects lost to follow-up between 2007 and 2011/2012 in the older age groups. However, especially in Senegal, and to a lesser extent in The Gambia, sustained circulation of ZIKV may have occurred between 2007 and 2011/2012 considering that some subjects negative in 2007 seroconverted in 2011/2012 and that some others positive in 2007 maintained or increased their antibody titers in 2011/2012. On the other hand, waning immunity occurred in few positive subjects in 2007 who became negative to ZIKV antibodies in 2011/2012. Evidence of decline in ZIKV naturally acquired neutralizing antibodies over time has been recently reported from French Polynesia and Fiji [27]. However, in our study 52.8% of subjects had maintained the same ZIKV antibody titer in 2011/2012, suggesting that natural immunity against ZIKV may persist for at least five years. In 2016, during the epidemic reported in French Guyana, vertical transmission of ZIKV has been estimated to occur in 26% of foetuses of ZIKV-infected mothers. Among foetuses exposed to ZIKV through vertical transmission, 20% had severe complications suggestive of congenital Zika syndrome [28]. Results from studies conducted since the mid-1950s have suggested that ZIKV has likely been endemic for decades in sub-Saharan Africa [29], with a recently documented severe outbreak in Cape Verde, Guinea Bissau and Angola. To date, only one documented case of ZIKV congenital syndrome from the Asian lineage has been reported, identified in a child from Portugal, in which the virus was imported from Angola [13], where mosquito-borne ZIKV transmission has been reported [14] together with an increased incidence of congenital microcephaly [30]. The lack of reports on ZIKV congenital syndrome in Africa may be explained either by the immunity acquired by women prior to childbearing age following ZIKV infection that results in protection against congenital infection [29], or by the fact that birth defects caused by ZIKV might have gone unnoticed and are often attributed to other pathogens [31].

It is of interest that in 2007, the first period of our study, ZIKV was reported in Micronesia [4] from where it spread to other Pacific Islands [32,33]. Phylogenetic studies have shown that the ZIKV outbreak in Micronesia was initiated by a strain from Southeast Asia, revealing the existence of an Asian lineage of ZIKV, different from the African one [34,35]. As the ZIKV Asian lineage was introduced into Africa only in 2015 [1], we can assume that between 2007 and 2012, only ZIKV African lineage was circulating in our study sites.

Recent “in vitro” and non-human primate model studies demonstrated that immunity after infection with one ZIKV lineage provides cross-protection against another [36,37]. However, little is known regarding any differences between the African and Asian lineages in clinical manifestations as well as on vertical transmission and ZIKV congenital syndrome, although some findings suggest that the Asian lineage might cause less severe disease [38]. Given that both lineages are now circulating in Africa, there is a growing need for a better understanding of the current epidemiology and public health impact of ZIKV in Africa.

## Figures and Tables

**Table 1:** Samples collected in Mali, Senegal and The Gambia in 2007 and 2011/2012 by age group and sex (%).

Study Site		Bamako, Mali		Niakhar, Senegal		Basse Santa Su, The Gambia	
Year(s) of Collection		2007	2011/2012	2007	2011/2012	2007	2011/2012
Age	2–10 y	97 (33.3)	86 (37.7)	86 (30.3)	87 (41.2)	98 (33.1)	83 (39.3)
	11–17 y	94 (32.3)	73 (32.0)	102 (35.9)	75 (35.5)	99 (33.4)	66 (31.3)
	18–29 y	100 (34.4)	69 (30.3)	96 (33.8)	49 (23.2)	99 (33.4)	62 (29.4)
Sex	M	171 (58.8)	128 (56.1)	158 (55.6)	111 (52.6)	179 (60.5)	128 (60.7)
	F	120 (41.2)	100 (43.9)	126 (44.4)	100 (47.4)	117 (39.5)	83 (39.3)
Total		291	228	284	211	296	211

**Table 2:** Anti-ZIKV microneutralization test (MNT) antibody prevalence in 2007 and in 2011/2012 in Mali, Senegal and The Gambia, by age group and sex (%, 95%CI).

Study Site		Bamako, Mali		Niakhar, Senegal		Basse Santa Su, The Gambia	
Year of Collection		2007 %(95%CI)	2011/2012 % (95%CI)	2007 % (95%CI)	2011/2012 % (95%CI)	2007 % (95%CI)	2011/2012 % (95%CI)
Age	2–10 y	0.0 (0.0–3.73)	0.0 (0.0–4.2)	1.2 (0.03–6.31)	2.3 (0.28–8.06)	0.0 (0.0–3.69)	0.0 (0.0–4.35)
	11–17 y	0.0 (0.0–3.85)	0.0 (0.0–4.93)	15.7 (9.24–24.22)	22.7 (13.79–33.79)	2.0 (0.25–7.11)	7.6 (2.51–16.8)
	18–29 y	1.0 (0.03–5.45)	1.4 (0.04–7.81)	21.9 (14.08–31.47)	20.4 (10.24–34.34)	9.1 (4.24–16.56)	16.1 (8.02–27.67)
Sex	M	0.6 (0.01–3.22)	0.8 (0.02–4.28)	12.0 (7.4–18.14)	11.7 (6.39–19.19)	3.4 (1.24–7.15)	7.8 (3.81–13.9)
	F	0.0 (0.0–3.03)	0.0 (0.0–3.62)	15.1 (9.33–22.54)	16.0 (9.43–24.68)	4.3 (1.4–9.69)	6.0 (1.98–13.5)
Total		0.3 (0.01–1.90)	0.4 (0.01–2.42)	13.4 (9.65–17.9)	13.7 (9.4–19.14)	3.7 (1.87–6.55)	7.1 (4.03–11.45)

**Table 3:** Anti-ZIKV MNT seroconversions in samples collected in Mali, Senegal and The Gambia, by age group and sex (*n*, %, 95%CI).

Study Site		Bamako, Mali *n* (%, 95% CI)	Niakhar, Senegal *n* (%, 95% CI)	Basse Santa Su, The Gambia *n* (%, 95% CI)
Age	2–10 y	0 (0.0, 0.0–4.35)	1 (1.4, 0.03–7.30)	0 (0.0, 0.0–4.45)
	11–17 y	0 (0.0, 0.0–5.21)	6 (8.0, 2.99–16.60)	3 (4.5, 0.95–12.71)
	18–29 y	1 (1.4, 0.04–7.81)	1 (2.0, 0.05–10.85)	3 (4.9, 1.03–13.71)
Sex	M	1 (0.8, 0.02–4.48)	5 (4.8, 1.58–10.86)	5 (4.0, 1.31–9.09)
	F	0 (0.0, 0.0–3.66)	3 (3.2, 0.66–9.04)	1 (1.2, 0.03–6.53)
Total		1 (0.5, 0.01–2.50)	8 (4.0, 1.76–7.81)	6 (2.9, 1.07–6.17)

**Table 4:** Anti-ZIKV MNT immunity over time in samples collected in Senegal and The Gambia, by MNT titer dynamics. The MNT titer range considered was 1:40–1:640 serum dilution. * included 6 samples from Senegal found negative in 2011/2012.

Study Site	Niakhar, Senegal *n*	Basse Santa Su, The Gambia *n*	Total *n* (%, 95% CI)
Same MNT titer	15	4	19 (52.8, 37.0–68.02)
Increase	4	3	7 (19.4, 9.45–35.33)
Decrease	8	2	10 * (27.8, 15.7–44.14)
Total	27	9	36
